# Literature-Informed Analysis of a Genome-Wide Association Study of Gestational Age in Norwegian Women and Children Suggests Involvement of Inflammatory Pathways

**DOI:** 10.1371/journal.pone.0160335

**Published:** 2016-08-04

**Authors:** Jonas Bacelis, Julius Juodakis, Verena Sengpiel, Ge Zhang, Ronny Myhre, Louis J. Muglia, Staffan Nilsson, Bo Jacobsson

**Affiliations:** 1 Department of Obstetrics and Gynecology, Sahlgrenska University Hospital Östra, Gothenburg, Sweden; 2 Department of Obstetrics and Gynecology, Institute of Clinical Sciences, Sahlgrenska Academy, University of Gothenburg, Gothenburg, Sweden; 3 Human Genetics Division, Cincinnati Children's Hospital Medical Center, Cincinnati, Ohio, United States of America; 4 Center for Prevention of Preterm Birth, Perinatal Institute, Cincinnati Children’s Hospital Medical Center and March of Dimes Prematurity Research Center Ohio Collaborative, Cincinnati, Ohio, United States of America; 5 Department of Genetics and Bioinformatics, Area of Health Data and Digitalisation, Norwegian Institute of Public Health, Oslo, Norway; 6 Department of Mathematical Sciences, Chalmers University of Technology, Gothenburg, Sweden; National Institute of Environmental Health Sciences, UNITED STATES

## Abstract

**Background:**

Five-to-eighteen percent of pregnancies worldwide end in preterm birth, which is the major cause of neonatal death and morbidity. Approximately 30% of the variation in gestational age at birth can be attributed to genetic factors. Genome-wide association studies (GWAS) have not shown robust evidence of association with genomic loci yet.

**Methods:**

We separately investigated 1921 Norwegian mothers and 1199 children from pregnancies with spontaneous onset of delivery. Individuals were further divided based on the onset of delivery: initiated by labor or prelabor rupture of membranes. Genetic association with ultrasound-dated gestational age was evaluated using three genetic models and adaptive permutations. The top-ranked loci were tested for enrichment in 12 candidate gene-sets generated by text-mining PubMed abstracts containing pregnancy-related keywords.

**Results:**

The six GWAS did not reveal significant associations, with the most extreme empirical *p* = 5.1 × 10^−7^. The top loci from maternal GWAS with deliveries initiated by labor showed significant enrichment in 10 PubMed gene-sets, e.g., *p* = 0.001 and 0.005 for keywords "uterus" and "preterm" respectively. Enrichment signals were mainly caused by infection/inflammation-related genes *TLR4*, *NFKB1*, *ABCA1*, *MMP9*. Literature-informed analysis of top loci revealed further immunity genes: *IL1A*, *IL1B*, *CAMP*, *TREM1*, *TFRC*, *NFKBIA*, *MEFV*, *IRF8*, *WNT5A*.

**Conclusion:**

Our analyses support the role of inflammatory pathways in determining pregnancy duration and provide a list of 32 candidate genes for a follow-up work. We observed that the top regions from GWAS in mothers with labor-initiated deliveries significantly more often overlap with pregnancy-related genes than would be expected by chance, suggesting that increased sample size would benefit similar studies.

## Introduction

The timing of human parturition is a poorly understood phenotype [1]. In the United States the reported rate of preterm birth (PTB), defined as birth occurring at less than 37 completed weeks of gestation, is 9.6% [2]. Worldwide PTB rates range from about 5% in some Northern European countries to 18% in Malawi [3]. PTB is the leading cause of death among neonates [4]. According to a US report, preterm born infants have a 15-fold higher mortality rate than those born at term [4]. More than 50% of deaths are attributable to only 2% of all infants—the ones who are born at less than 32 weeks of gestation [4]. PTB is also correlated with long-term adverse health consequences such as cerebral palsy, mental retardation, autism and schizophrenia, conditions that render individual dependent on the healthcare system. More than 50% of PTB occur in pregnancies without known risk factors. The currently available means of prediction (epidemiology- and biomarker-based models) and prevention (tocolytics, antibiotics, progesterone) are not effective enough to substantially reduce the rates of PTB and its adverse consequences [5].

Approximately 85% of all pregnancies have a spontaneous onset of delivery, with gestational age not affected by doctor’s decision to induce birth or to perform an elective caesarean section [6]. These pregnancies can be used for analysis of genetic factors affecting gestational age.

Up to 30% of variation in human gestational age could be accounted for by genetic factors, as reported by large register-based studies [7, 8]. The evidence of an acting genetic component motivated two genome-wide association studies (GWAS). In 2013 Uzun et al. [9] explored maternal genomes (884 preterm cases, 960 term controls). In 2015 Zhang et al. [10] investigated maternal (935 preterm cases, 946 term controls) and neonatal genomes (916 preterm cases, 935 term controls). The authors did not find robust statistical evidence of association between PTB and the 560 000 and 800 000 (respectively) single-nucleotide polymorphisms (SNPs) tested.

The failure to identify genes increasing the risk for PTB could be due to insufficient sample size, however it could also be due to the following problems: 1) preterm birth status has a lower information content than gestational age; 2) low accuracy of gestational age dating; 3) different onsets of delivery might reflect different aetiologies; 4) omission of genetic variants with low minor-allele frequency from analyses; 5) omission of non-additive genetic models in analyses; 6) mixed ethnicities in a study sample; 7) omission of prior knowledge about SNP function and the biological role of implicated genes. In our study we tried to avoid these shortcomings.

The aim of the study was to find SNPs that are associated with gestational age at birth. The use of gestational age, as opposed to the use of dichotomous PTB, provides an advantage, as it utilizes the full information present in the phenotype [11]. Our secondary aim was to highlight the genes that might mediate discovered associations, by identifying common biochemical pathways, networks, and functional similarities between the top genes. In the broadest sense, our study aims to account for a part of heritability of human gestational age at birth.

We structured our GWA study into six parts: investigating each of the subtypes (labor-initiated / PROM-initiated deliveries) separately and also together, while analysing maternal and fetal genomes separately.

## Methods

### The Dataset

#### Study population

The Norwegian Mother and Child Cohort (MoBa) is a nationwide pregnancy cohort managed by the Norwegian Institute of Public Health [12]. It includes more than 107 000 pregnancies recruited from 1999 through 2008. Most of the pregnant women in Norway received a postal invitation in connection to the routine ultrasound screening at gestational weeks 17–19. Participation rate was 42.7%. For the current study, individuals were sampled from the Version 4 database containing 71 669 pregnancies. The MoBa dataset is linked to the Medical Birth Registry of Norway (MBRN), for additional information see [13].

For genotyping we selected mothers and live-born children from singleton pregnancies of mothers in the age group of 20–34 years resulting in a spontaneous onset of delivery. Pregnancies with complications (e.g., preeclampsia, gestational diabetes, placental abruption, placenta previa, cervical cerclage, small for gestational age, fetal malformation), pregnancies of mothers with pre-existing medical conditions (e.g., diabetes, hypertension, inflammatory bowel disease, systemic lupus erythematosus, rheumatoid arthritis), as well as pregnancies conceived by in vitro fertilization were excluded [14]. Random sampling was done from two gestational age ranges: 154–258 days (preterm births) and 273–286 days (term births), thus creating an oversampling of lower gestational ages (**S1 Fig**). In total 1921 mothers and 1199 children were selected for genotyping using blood-extracted DNA. All mothers gave a written consent to use anonymised data in scientific research. The Norwegian Regional Ethics Committee for Medical Research approved the study (REK/Sør-Øst 2010/2683 S-6075).

#### Phenotype and covariates

**Fig 1:**
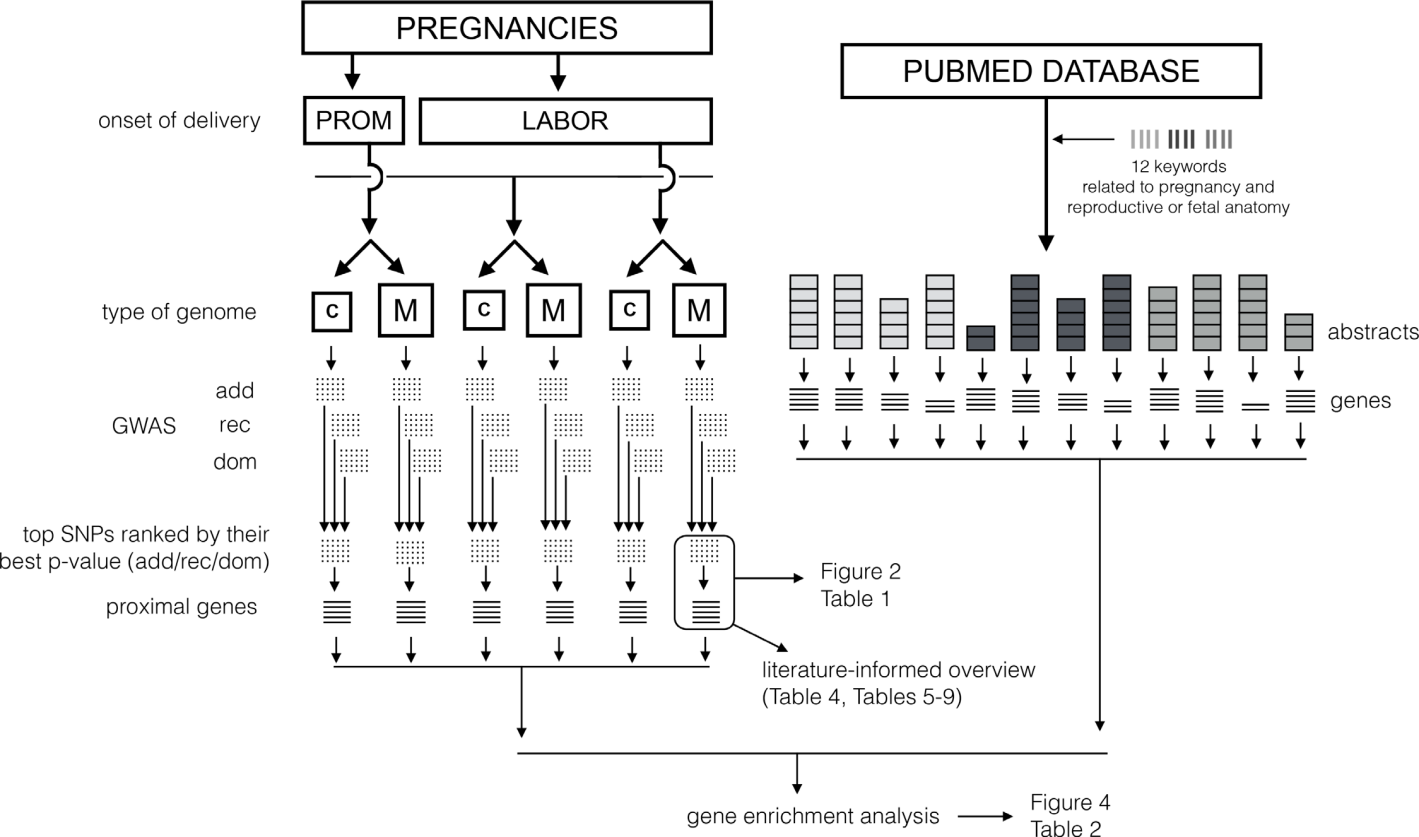
Schematic overview of the workflow in analyses. *C*—child genomes, *M—*maternal genomes; *add/rec/dom—*additive, recessive and dominant genetic models respectively.

We used gestational age expressed in days as a dependent variable, as continuous phenotype contains more information than a dichotomous case/control classification. MBRN provides an accurate second-trimester ultrasound-based evaluation of gestational age. Pregnancies initiated by labor were analysed separately from pregnancies starting with prelabor rupture of membranes (PROM), with one additional analysis where all pregnancies were considered together (**Fig 1**).

We also used non-genotyped MoBa cohort data with more than 70 000 pregnancies to evaluate potential impact of known covariates and risk factors on gestational age. Together, the evaluated covariates explained only 1% of variation in the continuous phenotype. Since 22.8% of genotyped individuals did not have values for some of these covariates, we decided to use the larger sample of genotyped individuals and not to use adjustment.

#### Genotyping quality control

The genotype missingness filter for SNPs and individuals was set to 3%. Individuals with heterozygosity estimates deviating by more than 3 SD from the group mean were removed. For each mother-mother or child-child pair related closer than second cousins, a random individual was removed. Hardy-Weinberg filter removed SNPs with *p* < 10^−6^. Non-Europeans were excluded after principal components analysis using the first three principal components and a threshold of 10 SD on the Euclidean distance from CEU cluster (HapMap). No minor-allele frequency filter was applied. Genomic inflation factor was estimated following standard procedures using continuous unadjusted gestational age in maternal samples (restricted to labor-initiated deliveries), linear regression, additive genetic model and minor-allele frequency restriction to > 0.06. All genomic positions are presented in hg19 coordinates.

### Association tests

Three genetic models (additive, recessive, dominant) were used to test for association with unadjusted continuous gestational age expressed in days (**Fig 1**). Permutation procedures are essential to avoid biases introduced by skewed phenotype distribution (a notable feature of gestational age), and by low counts of individuals in the minor genotypic group. We used permutation-based testing implemented in PLINK (v1.90b2n, 64-bit, 2 Nov 2014, Linux), with parameters for adaptive permutations: *alpha* = 5×10^−8^, *beta* = 5×10^−8^, min = 10, *max* = 1×10^9^, *b* = 1 and *a* = 0.001 [15]. Each SNP was assigned the most extreme empirical p-value from the three genetic models [16]: additive, recessive and dominant. X chromosomal SNPs were tested using only additive model. Two separate studies investigated our dataset for PTB association with X chromosomal SNPs [14] and mitochondrial SNPs [17] previously.

### Gene-set enrichment analysis with INRICH

#### Clumping

To merge adjacent and correlated SNPs, PLINK function “—clump” was used. Clumps were formed around “index variants” with p-value < 0.0005. Index variants were chosen greedily starting with the lowest p-value. Sites that were less than 250 kb away from an index variant, had r^2^ larger than 0.25 with it, and had association p-value smaller than 0.05 were assigned to that index variant's clump. The r^2^ values were computed using founders in the same genomic data.

#### PubMed gene-sets

We checked if the top GWAS loci were enriched in genes with known relations to pregnancy or reproductive anatomy. To test this, we used 12 *keywords* to create 12 gene-sets by text-mining the PubMed database, as described in the next paragraph and **Fig 1**. Out of these, 4 keywords represent pregnancy conditions (“gestation”, “parturition”, “pregnancy”, “preterm”), another 4 describe female anatomy (“cervix”, “endometrium”, “myometrium”, “uterus”), and the last 4 portray fetal anatomy (“fetus/embryo”, “chorion”, “amnion”, “placenta”). We also created 16 gene-sets for *keywords* unrelated to pregnancy to be used as a control in enrichment analysis: 8 representing conditions and 8 representing anatomy **(S1 Table)**.

Between June 1st and August 31st, 2015, the PubMed database was scanned for abstracts containing any semantic form or Latin/Greek form of the selected *keyword* together with the words indicating the genetic nature of a publication (“gene”, “genes”, “genomic”, “genetic” or “GWAS”; plus corresponding MeSH terms), but restricted to abstracts not containing 65 custom-built non-human subject indicators (e.g., “cat”, “feline”, “cow”, “bovine”) or 466 custom-built medical-condition indicators (e.g., listeriosis, erythema, hepatitis, neuroblastoma). The latter indicators were constructed by text-mining the ICD code database (www.cms.gov) and searching for words with common disease suffixes (e.g., "-osis", "-itis", "-emia", "-oma"). These restrictions were applied to avoid inclusion of genes that represent medical conditions or species not present in our GWAS data. Abstracts were mined searching for gene names by cross-referencing each capitalised word with 23 945 HGNC [18] gene names. We took precaution to avoid false identification of commonly used acronyms as gene names, e.g., gene *AGA* and “Apropriate for Gestational Age”, gene *FGR* and “Fetal Growth Retardation”, gene *SPTB* and “Spontaneous Preterm Birth”. In order to further reduce erroneous assignment of genes to *keywords*, only the genes mentioned in more than 1 abstract were used. In order to obtain a better representation of the *keyword*, we also used an "exclusivity" filter: the abstract must not contain more than one different *keyword* (with exception for very common and control *keywords*). All *keywords* and PubMed queries are listed in **S1 Table**.

#### Enrichment analysis

Each clump produced by PLINK represents a genomic region defined by distance, linkage disequilibrium (LD) and statistical association with the phenotype. INRICH [19] is a tool that detects overlap between such regions and predefined gene sets and reports the empirically estimated p-value of enrichment. For this purpose INRICH iteratively generates random clumps of similar size and SNP-density and then creates a distribution of enrichment statistic under the null-hypothesis (“no enrichment”). P-values estimated with this method are expected to be robust and unbiased. Analysis was performed using the INRICH algorithm (v.1.0, updated Oct/24/2014, Linux). GWAS interval was considered to be a 'hit' for a predefined gene-set if it fell within 25 kb of any of the genes in that set, 100 000 permutations were used to estimate p-values for each gene-set, maintaining 90–110% SNP density match. The 300 top clumps from each of the six GWAS (mothers, children × labor, PROM, all) were tested against 12 pregnancy-related gene-sets and 16 control gene-sets from the PubMed abstract mining (**Fig 1**).

#### Literature-informed analysis of GWAS results

By manually cross-referencing the 300 top SNPs from maternal GWAS in labor-initiated deliveries with the HaploReg v4.1 database (www.broadinstitute.org/mammals/haploreg, [20]) and with the scientific publication database MEDLINE, we selected biologically-relevant SNPs with their implicated genes. We grouped genes into categories, based on biological pathway that could modify gestational age. A prior evidence of association with gestational age / preterm birth, or evidence of interaction or functional/structural similarity among the top genes were used as the criteria for reporting genes in the result tables.

## Results

### Genotyping quality control

After quality control procedures of genotyping data, 1743 maternal and 1109 fetal samples were left and had relevant phenotypic data (1407 labor and 336 PROM mothers; 884 labor and 225 PROM children). The number of genotyped SNPs passing the quality-control procedures is 513 273 autosomal and 12 304 from the X chromosome. Mitochondrial, Y chromosomal SNPs and pseudo-autosomal SNPs were not analysed in this study. Principal components analysis of genotyping data assured that study individuals belong to a homogeneous European population. Geographical homogeneity was also reflected by genomic inflation factor, estimated to be 0.993 and indicating no population stratification effects in GWAS for this phenotype.

### Association tests

**Fig 2:**
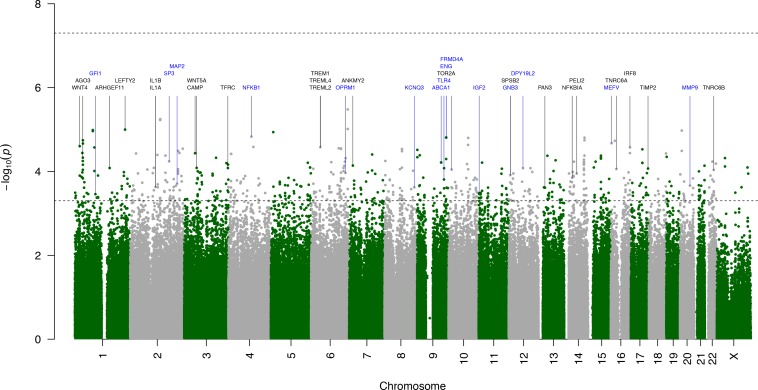
Manhattan plot for maternal GWAS of gestational age in labor-initiated deliveries. In total 1 407 genomes were analysed. Each SNP was assigned the most extreme empirical p-value from the three genetic models (additive, recessive, dominant). The top line indicates a genome-wide significance level (5×10^−8^), while the bottom line marks a significance level (5×10^−4^) determining the number of “clumps” (independent loci that were used in gene-set enrichment analyses). Genes from gene-set enrichment analyses are marked in blue, while other biologically relevant genes (from the literature-informed analyses) are marked in black.

None of the 525 577 SNPs tested with the additive, recessive and dominant genetic models showed a genome-wide significance (*p* < 5×10^−8^) in any of the six GWA analyses. The most extreme association was observed in a GWAS with PROM mothers (*p* = 5.1 × 10^−7^, SNP rs6977715 in the *DPP6* gene). Due to the further-described findings in the post-GWAS analysis, in **Fig 2** we present only the results from a GWAS of maternal genomes and labor-initiated deliveries, with the top 20 independent loci together with proximal genes highlighted in **Table 1**. The top results from the remaining GWA analyses are presented in **S2 Fig** and **S1 File**.

**Table 1:** Top 20 independent loci from maternal GWAS of gestational age in labor-initiated deliveries.

Chr	BP	SNP	P	E/R	Mod	nEE	nER	nRR	mEE	mER	mRR	Genes
6	164389165	rs593254	3.32e-6	A/G	ADD	76	480	851	260	264	268	
2	134837980	rs13410504	5.64e-6	G/A	REC	7	237	1163	221	265	267	
1	226209989	rs17515010	1.00e-5	G/A	REC	4	141	1261	205	264	266	*SDE2*, *PYCR2*, ***LEFTY2***, *H3F3A*, *H3F3AP4*
1	81391541	rs17105699	1.03e-5	G/A	DOM	6	165	1232	273	259	267	
20	7618077	rs6086132	1.06e-5	A/G	DOM	162	649	596	269	268	263	
5	10501076	rs2589658	1.15e-5	C/A	REC	330	684	393	262	267	267	*ROPN1L*, *ROPN1L-AS1*, *MARCH6*, *ANKRD33B*
4	103537442	rs1609798	1.48e-5	A/G	REC	128	601	677	259	266	268	***NFKB1***, *MANBA*
9	130417033	rs10117075	1.55e-5	A/G	REC	12	190	1205	237	268	266	*TTC16*, ***TOR2A***, *STXBP1*, *SH2D3C*, *PTRH1*, *FAM129B*
14	91352234	rs6575165	1.56e-5	A/G	ADD	87	478	842	260	264	268	*TTC7B*, *RPS6KA5*
10	88336279	rs2588278	1.58e-5	A/G	ADD	260	680	467	270	266	264	*WAPAL*, *OPN4*, *LDB3*
1	36879232	rs3007217	1.81e-5	G/A	ADD	150	593	664	270	268	264	*STK40*, *SH3D21*, *OSCP1*, *MRPS15*, *LSM10*, *EVA1B*, *CSF3R*
16	18067234	rs151699	1.86e-5	C/A	REC	1	133	1272	161	266	266	
16	3344618	rs220381	2.12e-5	G/A	DOM	159	559	689	270	268	264	*ZSCAN32*, *TIGD7*, *OR2C1*, *OR1F2P*, *OR1F1*, *MTRNR2L4*, ***MEFV***
10	87762136	rs11201867	2.33e-5	A/G	ADD	22	304	1081	275	270	265	*GRID1*
1	22345093	rs3117048	2.49e-5	A/G	REC	146	633	628	273	266	265	***WNT4***, *HSPG2*, *CELA3B*, *CELA3A*, *CDC42*
4	112524778	rs10015214	2.60e-5	A/G	DOM	312	675	420	266	268	263	
6	41164005	rs6915083	2.64e-5	G/A	REC	197	648	561	261	268	266	TREM*, TREML*, *NFYA*, *ADCY10P1*
16	85941774	rs305080	2.67e-5	A/G	REC	143	586	678	273	266	265	***IRF8***
1	88453303	rs3008465	2.67e-5	C/A	ADD	71	532	802	272	268	264	
6	123749752	rs1343962	2.80e-5	A/G	ADD	303	713	390	263	266	269	*TRDN*

Table 1 was pruned to show only independent loci. *BP*—physical position on the chromosome in hg19 coordinates, *P—*the most extreme empirical p-value from three genetic models, *E/R—*the effect allele and the reference allele, *Mod*—the most significant genetic model for that SNP, *nXX*—number of individuals in each genotypic group, *mXX*—mean gestational age in each genotypic group. Interpretation of mean gestational age values should take into account the bimodal phenotype distribution of genotyped individuals (**S1 Fig**). *Genes* were assigned to SNPs based on a 100 kb offset rule. Asterisk (*) indicates a gene family with multiple genes in that locus. No multiple-test correction is applied. Bolded genes are described in the literature-informed analyses. Genes with unknown function (*LINC*, *LOC* etc.) are not listed.

**Fig 3:**
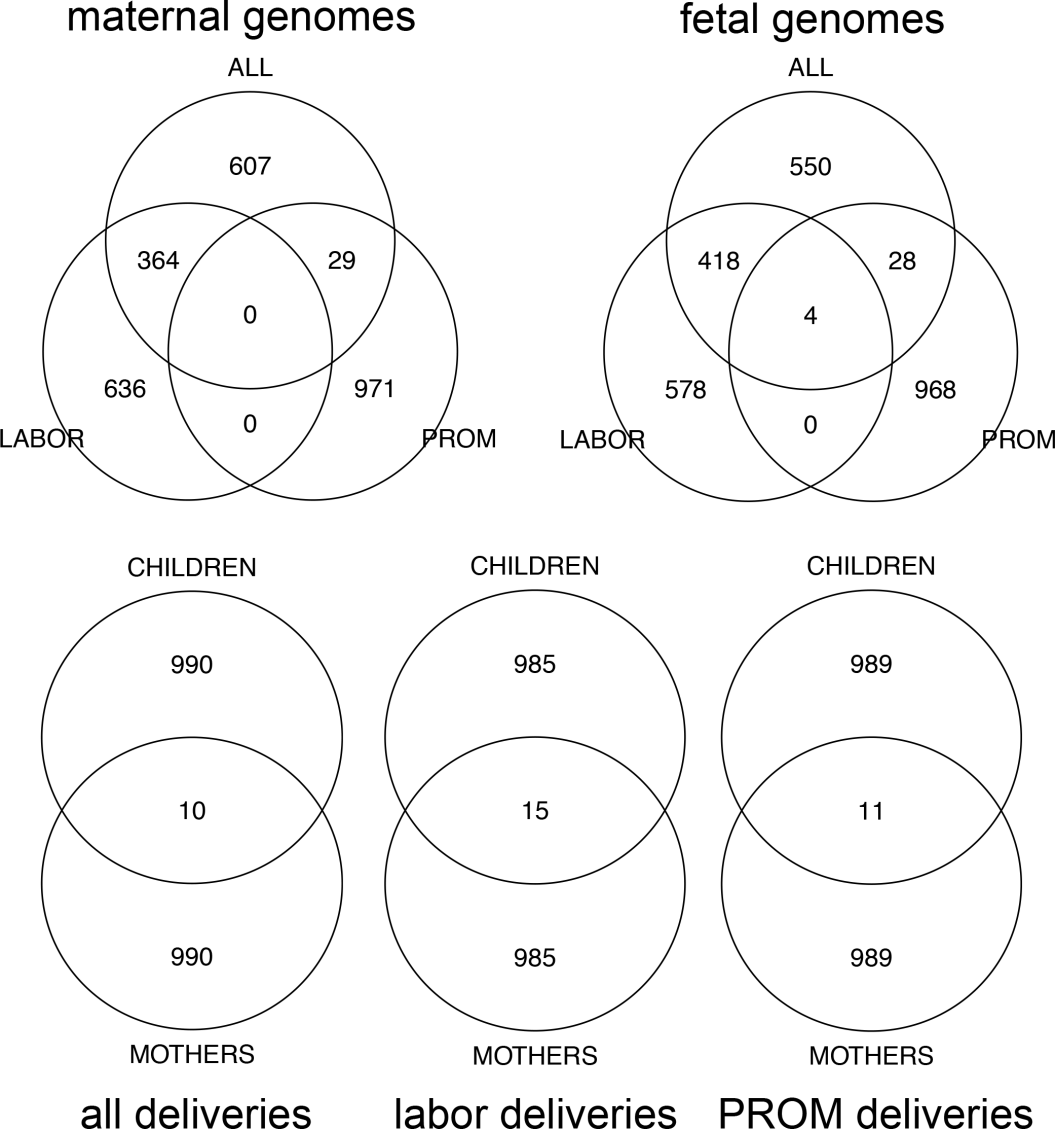
Overlap between top results in six GWAS. The top 1000 SNPs were selected from each GWA analysis. Numbers in the Venn diagrams represent the number of SNPs. Numbers of individuals in each analysis were 1743, 1407, 336 (mothers) and 1109, 884, 225 (children) for all together, labor-initiated and PROM-initiated deliveries respectively.

The GWAS with labor-initiated deliveries and the GWAS with all deliveries shared approximately one-third and one-half of the top SNPs in maternal and fetal genomes respectively, while top SNPs from GWAS with PROM-initiated deliveries were mostly unique **(Fig 3)**.

### Gene-set enrichment analysis with INRICH

#### Gene-sets

The sizes of the gene-sets in the PubMed-constructed pregnancy-themed group are as follows: 123 “preterm” genes, 214 “gestation” genes, 20 “parturition” genes, 540 “pregnancy” genes; maternal anatomy group: 59 “cervix” genes, 116 “endometrium” genes, 23 “myometrium” genes, 74 “uterus” genes; fetal anatomy group: 14 “fetus/embryo” genes, 35”chorion” genes, 45 “amnion” genes, 259 “placenta” genes. The full list of gene-set sizes with respective PubMed queries is shown in **S1 Table**. The full list of genes in each set is given in the **S2 File**.

#### Enrichment analysis

**Fig 4:**
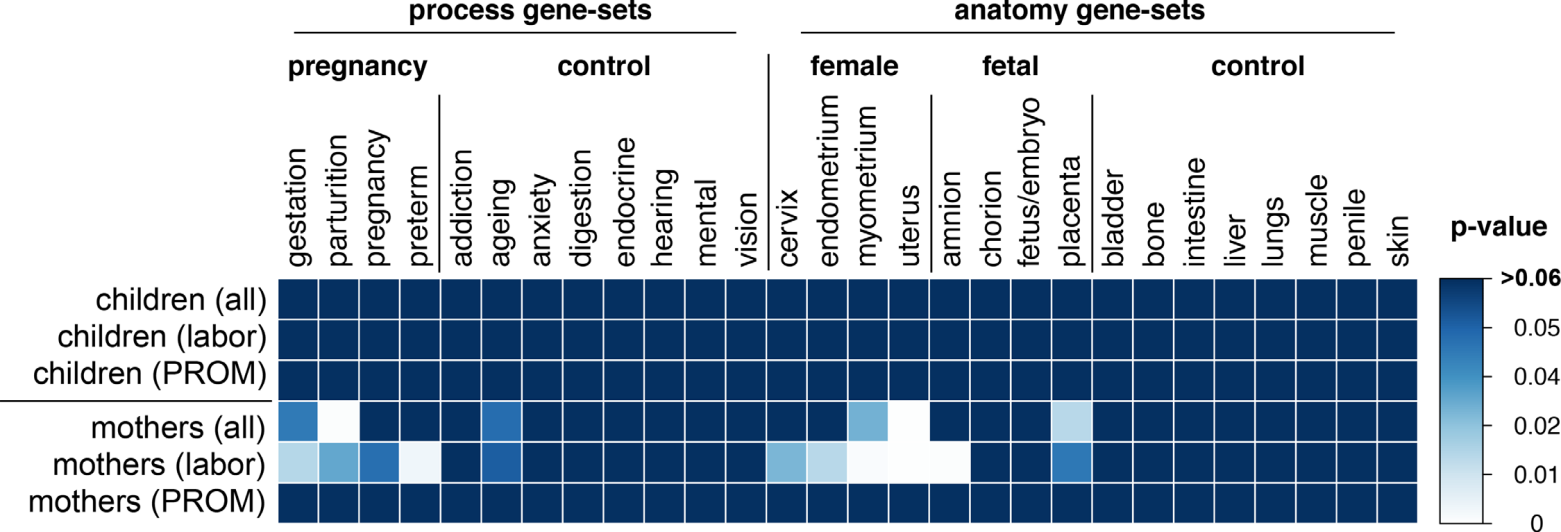
Enrichment in gene-sets generated using PubMed abstract text-mining. The figure shows an overlap between the genes implicated in six GWA analyses (rows) and genes related to specific *keywords* (columns). The overlap is represented as probability (p-value) of similar or greater enrichment arising due to pure chance under the null hypothesis of no enrichment (i.e., if GWAS would rank genes in a random order). The 300 top independent loci (“clumps”) and their genes were used. The name of each gene-set indicates a *keyword* used in the PubMed abstract mining. The INRICH algorithm was used to estimate empirical p-values.

Only the maternal GWAS with labor-initiated deliveries showed consistent enrichment in all relevant candidate gene-sets, and consistently showed no enrichment in the control gene-sets (**Fig 4**).

In this particular analysis (mothers with labor-initiated deliveries), out of 300 selected top GWAS clumps, the INRICH algorithm removed 116 intervals without genes and then merged some of the remaining to form a final number of 178 independent (non-overlapping) genomic intervals. The top GWAS genes overlapping with candidate gene-sets are presented in **Table 2** together with a probability of observing a similar or more extreme overlap under no genotype-phenotype association. The gene-set with the most significant enrichment corresponds to the keyword "uterus" (empirical *p* = 0.001). This gene-set contains 73 genes, 5 of which overlap with top GWAS intervals: *ENG* (endoglin), *IGF2* (insulin-like growth factor 2), *MMP9* (matrix metallopeptidase 9), *NFKB1* (nuclear factor κ-B DNA binding subunit), *TLR4* (toll-like receptor 4). These genes were also present in many other significantly enriched candidate gene-sets. **Table 3** shows SNPs that implicated genes from **Table 2**, together with p-values from maternal GWAS using labor-initiated deliveries and genomic coordinates of respective clumped regions. Only 1 out of 16 control gene-sets ("ageing") was enriched (*p* = 0.05), while 10 out of 12 candidate gene-sets were enriched: all 4 pregnancy-themed sets, all 4 female anatomy sets, and 2 out of 4 fetal anatomy sets.

**Table 2:** Significantly enriched PubMed gene-sets in GWAS using mothers with labor-initiated deliveries.

Gene set	N genes	Hits	P	Enriched genes
***Preterm***	123	6	0.005	*IGF2*, *KCNQ3*, *MMP9*, *NFKB1*, *OPRM1*, *TLR4*
***Gestation***	212	7	0.018	*ENG*, *IGF2*, *KCNQ3*, *MMP9*, *NFKB1*, *OPRM1*, *TLR4*
***Parturition***	20	2	0.031	*MMP9*, *NFKB1*
***Pregnancy***	536	12	0.046	*ABCA1*, *DPY19L2*, *ENG*, *FRMD4A*, *GFI1*, *GNB3*, *IGF2*, *KCNQ3*, *MEFV*, *MMP9*, *NFKB1*, *TLR4*
*Ageing*	76	3	0.049	*IGF2*, *MMP9*, *NFKB1*
***Cervix***	59	3	0.026	*MMP9*, *NFKB1*, *TLR4*
***Endometrium***	116	5	0.014	*IGF2*, *MMP9*, *NFKB1*, *SP3*, *TLR4*
***Myometrium***	23	3	0.002	*MMP9*, *NFKB1*, *TLR4*
***Uterus***	73	5	0.001	*ENG*, *IGF2*, *MMP9*, *NFKB1*, *TLR4*
***Amnion***	45	4	0.002	*IGF2*, *MAP2*, *MMP9*, *NFKB1*
***Placenta***	258	7	0.043	*ABCA1*, *ENG*, *IGF2*, *KCNQ3*, *MMP9*, *NFKB1*, *TLR4*

The column *N genes* indicates the number of genes in a gene-set, while *Hits* states how many overlap (25kb offset) with the genes from the top 300 independent GWAS loci ("clumps"). The empirical p-value of enrichment (*P*) is estimated using INRICH algorithm with 100 000 permutations. Only significantly enriched gene-sets (*p* < 0.05) are shown out of 12 candidate sets and 16 control sets tested. No multiple-test correction is applied.

**Table 3:** Genomic loci that implicate the genes mentioned in Table 2.

Rank	SNP	P	Clumped region	Gene
**7**	rs1609798	1.48e-5	chr4:103396333..103647047	*NFKB1*
**8**	rs10117075	1.55e-5	chr9:130358236..130586688	*ENG*
**13**	rs220381	2.12e-5	chr16:3301897..3344618	*MEFV*
**51**	rs6718188	5.73e-5	chr2:174739352..174835769	*SP3*
**57**	rs12336969	6.10e-5	chr9:107679500..107684276	*ABCA1*
**77**	rs1607800	8.36e-5	chr12:63790463..63982989	*DPY19L2*
**84**	rs3740121	9.01e-5	chr10:13834678..13838604	*FRMD4A*
**100**	rs12202611	1.08e-4	chr6:154204327..154333183	*OPRM1*
**114**	rs2301137	1.22e-4	chr12:6956462..7053149	*GNB3*
**142**	rs7045953	1.56e-4	chr9:120446826..120485795	*TLR4*
**169**	rs2365661	1.96e-4	chr2:210154210..210391837	*MAP2*
**187**	rs3746512	2.18e-4	chr20:44577314..44662413	*MMP9*
**211**	rs1457776	2.39e-4	chr8:133355244..133423654	*KCNQ3*
**285**	rs4320932	3.28e-4	chr11:2117403..2171601	*IGF2*
**295**	rs6662618	3.48e-4	chr1:92935411..93148377	*GFI1*

The *Rank* represents a rank of an independent genomic region ("clump") based on the most extreme GWAS p-value (*P*) of the representative index SNP in three genetic models. Genomic positions of regions are presented in hg19 coordinates.

### Literature-informed analysis of GWAS results

Manual inspection of the top 300 SNPs from maternal GWAS in labor-initiated deliveries highlighted 32 biologically relevant genes from 27 independent loci (**Table 4**). In total 284 genes had their biological background evaluated.

**Table 4:** Loci of biological relevance from maternal GWAS of gestational age in labor-initiated deliveries.

Rank	SNP	Chr	BP	P	E/R	Mod	nEE	nER	nRR	mEE	mER	mRR	Genes
**5**	rs17515010	1	226209989	1.00e-5	G/A	REC	4	141	1261	205	264	266	*LEFTY2*
**10**	rs1609798	4	103537442	1.48e-5	A/G	REC	128	601	677	259	266	268	*NFKB1*
**11**	rs10117075	9	130417033	1.55e-5	A/G	REC	12	190	1205	237	268	266	*ENG*
**13**	rs2287116	9	130420813	1.55e-5	A/C	REC	12	210	1185	237	267	266	*TOR2A*
**19**	rs220381	16	3344618	2.12e-5	G/A	DOM	159	559	689	270	268	264	*MEFV*
**22**	rs3117048	1	22345093	2.49e-5	A/G	REC	146	633	628	273	266	265	*WNT4*
**24**	rs6915083	6	41164005	2.64e-5	G/A	REC	197	648	561	261	268	266	*TREM1*, *TREML2*, *TREML4*
**25**	rs305080	16	85941774	2.67e-5	A/G	REC	143	586	678	273	266	265	*IRF8*
**41**	rs4312673	3	48401307	3.67e-5	A/G	DOM	1	72	1332	282	256	267	*CAMP*
**65**	rs634335	1	36335862	5.63e-5	C/A	DOM	23	310	1074	266	262	267	*AGO3*
**66**	rs6718188	2	174761611	5.73e-5	A/C	ADD	157	611	638	269	268	264	*SP3*
**75**	rs12336969	9	107679500	6.10e-5	A/C	REC	7	201	1199	229	267	266	*ABCA1*
**88**	rs2177539	7	16652523	7.24e-5	G/A	REC	109	566	728	259	267	267	*ANKMY2*
**98**	rs3913369	3	55481075	8.22e-5	A/C	ADD	69	498	840	262	264	268	*WNT5A*
**100**	rs12138039	1	156918137	8.29e-5	A/G	DOM	6	185	1214	259	261	267	*ARHGEF11*
**101**	rs4075688	3	195848264	8.30e-5	G/A	REC	177	668	559	261	266	268	*TFRC*
**106**	rs4789863	17	76897347	8.52e-5	A/G	DOM	1	122	1281	251	259	267	*TIMP2*
**109**	rs11866271	16	24881152	8.74e-5	C/A	DOM	107	582	713	266	264	268	*TNRC6A*
**117**	rs3021274	22	40395084	9.22e-5	A/G	DOM	230	653	524	269	267	264	*TNRC6B*
**138**	rs12202611	6	154237443	1.08e-4	G/A	REC	7	295	1105	230	266	266	*OPRM1*
**146**	rs395643	14	56541638	1.12e-4	G/A	REC	14	310	1083	242	266	266	*PELI2*
**157**	rs2301137	12	7018949	1.22e-4	A/G	DOM	86	536	784	267	263	268	*GNB3*, *SPSB2*
**173**	rs12435366	14	35838389	1.41e-4	A/G	REC	97	550	745	259	267	266	*NFKBIA*
**197**	rs7045953	9	120485795	1.56e-4	G/A	ADD	37	379	991	272	269	265	*TLR4*
**266**	rs3746512	20	44592636	2.18e-4	A/G	REC	34	394	979	253	266	267	*MMP9*
**284**	rs4849122	2	113560921	2.34e-4	G/A	REC	7	158	1242	233	266	266	*IL1A*, *IL1B*
**293**	rs1457776	8	133360660	2.39e-4	A/G	REC	52	433	922	256	266	267	*KCNQ3*
**299**	rs942364	13	28896097	2.44e-4	A/G	DOM	20	307	1080	270	262	267	*PAN3*

The SNPs were selected from the top 300 GWAS results, based on their proximity and/or functional relationship with genes biologically relevant to gestational age. *Rank—*the rank of that SNP among all GWAS results, based on the most significant empirical p-value (*P*) from three genetic models, *BP*—physical position on the chromosome (*Chr*) in hg19 coordinates, *E/R—*the effect allele and the reference allele, *Mod*—the most significant genetic model for that SNP, *nXX*—number of individuals in each genotypic group, *mXX*—mean of gestational age in each genotypic group. Interpretation of mean gestational age values should take into account the bimodal phenotype distribution of genotyped individuals (**S1 Fig**). No multiple-test correction is applied.

We grouped these genes into four functional categories related to possible aetiologies of preterm birth: 1) bacterial or viral infection 2) utero-placental perfusion problems 3) cervical insufficiency 4) hormonal imbalance.

#### Infection

Bacterial infection is a well-known cause of too short gestation [1]. We observed 14 SNPs that are known expression quantitative trait loci (eQTLs) for (or are located in proximity of) 17 immunity-related genes (**Table 5**). Most of these genes act through activation of nuclear factor complex NF-κB, a central regulator of the terminal processes in human labor and delivery [21].

**Table 5:** An overview of infection-related genes.

SNP	Rank	*p*-value	Gene	Function / relevance
**rs1609798**	10	1.5e-5	***NFKB1***	SNP is an eQTL for nuclear factor NFKB1 [22] known for association with preterm birth [23].
**rs220381**	19	2.1e-5	***MEFV***	SNP is an eQTL for pyrin (marenostrin) encoded by *MEFV* [22]. Pyrin is an important modulator of innate immunity [24]. As a regulator of IL1B activation, pyrin might be involved in preterm birth, especially after intrauterine infection [25].
**rs6915083**	24	2.6e-5	***TREML2*, *TREM1*, *TREML4***	SNP is in LD (r^2^ = 0.7) with a missense mutation in *TREML2*. This mutation (rs3747742) is also an eQTL for immunoreceptor encoded by a proximal gene *TREM1* [22], which amplifies responses to bacterial lipopolysaccharide and is elevated in the cord blood of preterm fetuses [26]. Mutation is also an eQTL for a proximal gene *TREML4 [22]*, which is a positive regulator of TLR7 signalling responsible for detecting single-stranded viral RNA [27].
**rs305080**	25	2.7e-5	***IRF8***	SNP is an eQTL for interferon regulatory factor encoded by *IRF8* [22]. Importantly, interferon-γ protein is associated with preterm birth [28, 29], while SNP in interferon-γ gene is also associated with preterm birth [30].
**rs4312673**	41	3.7e-5	***CAMP***	A proximal gene *CAMP* encodes cathelicidin antimicrobial peptide, which binds to bacterial lipopolysaccharides and regulates inflammatory response. CAMP is present in the first trimester cervicovaginal secretions and is expressed at higher levels in women with bacterial vaginosis [31]. CAMP levels are higher in foetal membranes and myometrium after spontaneous labour than after elective caesarean section [32]. The SNP is also an eQTL for a proximal gene *ZNF589* [33], which forms a fusion gene with *CAMP*.
**rs12336969**	75	6.1e-5	***ABCA1***	Intronic SNP in *ABCA1* gene. Maternal expression of *ABCA1* was previously associated with decreased gestational age [34]. This relation could be explained by ABCA1 involvement in infection-response [35]. Interestingly, a short-half-life ABCA1 protein binds to ARHGEF11 (**Table 7**), which prevents ABCA1 degradation [36].
**rs3913369**	98	8.2e-5	***WNT5A***	SNP is the most proximal to *WNT5A* gene and is in LD with 3'-UTR variant (r^2^ = 0.9). *WNT5A* is upregulated under bacterial infection via TLR4 and NFKB activation, which induces interferon-γ production [37]. Lipopolysaccharide enhances *WNT5A* expression through TLR4 and NF-κB pathways [38]. Interestingly, WNT5A induces expression of fibronectin [39], a marker for preterm birth [40].
**rs4075688**	101	8.3e-5	***TFRC***	SNP is an eQTL for transferrin receptor TFRC [22], which binds to iron-loaded transferrin and sequesters iron inside a cell via receptor-mediated endocytosis. This is the first line of defense against bacterial infection called "nutritional immunity" (bacterial pathogens are dependent on iron from their hosts) [41]. Concentrations of transferrin receptors are significantly increased in women with vaginal infection [42]. Similarly, elevated maternal serum ferritin (another iron-binding protein) concentrations are associated with preterm birth [43] and intrauterine growth restriction [44], possibly via similar defense mechanism.
**rs395643**	146	1.1e-4	***PELI2***	SNP is an eQTL for pellino protein [22] necessary for activation of NF-κB complex.
**rs2301137**	157	1.2e-4	***SPSB2***	SNP is an eQTL for SPSB2 protein [22], which is involved in infection defense via the nitric oxide production [45].
**rs12435366**	173	1.4e-4	***NFKBIA***	Proximal-gene product inhibits NFKB1 responses, also affects the expression of *TFRC*, as a defence-to-bacterial-infection strategy [41].
**rs7045953**	197	1.6e-4	***TLR4***	SNP is an eQTL for toll-like receptor TLR4 [22] that recognizes structurally conserved molecules derived from microbes. *TLR4* mRNA levels are significantly elevated in preterm-delivering women [46]. TLR4 plays a critical role in inflammation-induced preterm birth in a murine model [47].
**rs3746512**	266	2.2e-4	***MMP9***	SNP is an eQTL for extracellular matrix remodelling enzyme matrix metalloproteinase MMP9 [22]. A genetic variant in MMP9 promoter is associated with preterm birth [48]. In myometrium, bacterial fragments increase the expression of *MMP9* [21].
**rs4849122**	284	2.3e-4	***IL1A***, ***IL1B***	Interleukins IL1A and IL1B are mediators between infection and inflammation. Genetic variants in *IL1A* and *IL1B* were associated with preterm birth in [49] and [50] respectively.

Genes were selected from the top 284 genes (top 300 SNPs) in maternal GWAS with labor-initiated deliveries. The genes are presented together with the leading SNP from that region and its most extreme empirical *p-value* from three genetic models. *Rank* represents the rank of that SNP among all GWAS results.

Besides their individual connection to preterm birth via immunity mechanisms, ten genes from independent loci interact among each other: Pyrin encoded by *MEFV* decreases activation of NF-κB complex [24], which includes NFKB1; pellino protein encoded by *PELI2* is necessary for activation of NF-κB complex; NF-κB activation is induced by lipopolysaccharide and interleukine encoded by *IL1B*; NFKB1 binds with IRF8 [51]; CAMP decreases expression of *NFKB1* [52]; *NFKBIA* gene (independent region from NFKB1) product inhibits NFKB1 responses; NFKBIA affects the expression of *TFRC*, as a defence-from-bacterial-infection strategy [41]; IL1B increases *NFKBIA* expression; *SPSB2* gene together with the *MEFV* gene share a SPRY domain, which is involved in innate immunity [53]; IL1B can increase expression of *MMP9* [54].

Viral infection is also a potential cause of preterm birth [55]. In **Table 6** we present biologically relevant "viral-immunity" genes identified by maternal GWAS in labor-initiated deliveries. During the pregnancy, the immune system actively supports the growing fetus. Viral infection weakens this function allowing other microorganisms to propagate and lead to preterm birth [56]. Five genes are known to bind to each other and are likely to play a role in the defense against viral infection by utilizing the RNA-induced silencing complex (RISC). Argonautes (encoded by *AGO1*, *AGO3*, *AGO4*) are the main components of RISC together with TNRC6A and TNRC6B (both TNRC genes are located on different chromosomes). The host can inhibit viral replication using a library of miRNAs that matches parts of viral RNA [57], and a RISC complex [58]. Moreover, the ability to suppress RISC was suggested as a counter-strategy deployed by viruses [59]. The ribonuclease subunit encoded by *PAN3* binds to both TNRC proteins, while ANKMY2 binds to AGO3.

**Table 6:** An overview of "viral-immunity" genes.

SNP	Rank	*p*-value	Gene	Function / relevance
**rs634335**	65	5.6e-5	***AGO3***	SNP is an eQTL for a proximal gene *AGO3* [60], which is a component of RNA-induced silencing complex (RISC).
**rs2177539**	88	7.2e-5	***ANKMY2***	Intronic SNP in the gene *ANKMY2* encoding a protein, which binds to AGO3.
**rs11866271**	109	8.7e-5	***TNRC6A***	SNP is an eQTL for a proximal gene *TNRC6A* [22], which encodes a component of RISC complex.
**rs3021274**	117	9.2e-5	***TNRC6B***	The second most proximal gene encodes a component of RISC complex.
**rs942364**	299	2.4e-4	***PAN3***	SNP is an eQTL for a proximal gene *PAN3* [33].

Genes were selected from the top 284 genes (top 300 SNPs) in maternal GWAS with labor-initiated deliveries. The genes are presented together with the leading SNP from that region and its most extreme empirical *p-value* from three genetic models. *Rank* represents the rank of that SNP among all GWAS results.

#### Utero-placental perfusion problems

In **Table 7** we show the second group of genes that are involved in utero-placental perfusion problems characterised by either utero-placental angiogenic imbalances (*LEFTY2*, *ENG*, *KCNQ3*, *TIMP2*, *MMP9*, *ABCA1*), maternal blood pressure (*TOR2A*, *ARHGEF11*, *GNB3*), or by compromised placentation (*WNT4*, *WNT5A*).

**Table 7:** An overview of utero-placental perfusion genes.

SNP	Rank	*p*-value	Gene	Description
**rs17515010**	5	1.0e-5	***LEFTY2***	The third most proximal gene *LEFTY2* encodes a growth factor, an important member of the Nodal signalling pathway essential for uterine cycling, embryo implantation and endometrial decidualization [61].
**rs10117075**	11	1.6e-5	***ENG***	SNP is in LD (0.44 r^2^) with synonymous mutation in gene *ENG* encoding transforming growth factor component endoglin involved in angiogenesis and preeclampsia [62].
**rs2287116**	13	1.6e-5	***TOR2A***	SNP is an eQTL for a potent hypotensive peptide TOR2A [60], which stimulates the release of vasopressin [63] and is associated with impaired intrauterine growth [64].
**rs3117048**	22	2.5e-5	***WNT4***	SNP is located 99 kb from the *WNT4* gene. Wnt4 is important signalling molecule in decidualisation [65] in the mouse model.
**rs12336969**	75	6.1e-5	***ABCA1***	Intronic SNP in *ABCA1* gene. Maternal expression of *ABCA1* was previously associated with decreased gestational age [34], which could be explained by the fact that *ABCA1* is upregulated by hypoxia [66] and plays a critical role in proper angiogenesis [67]. Interestingly, a short-half-life ABCA1 protein binds to ARHGEF11 (see below), which prevents ABCA1 degradation [36].
**rs3913369**	98	8.2e-5	***WNT5A***	SNP is the most proximal to *WNT5A* gene and is in LD with 3'-UTR variant (r^2^ = 0.9). *WNT5A* encodes a major signalling molecule critical to healthy embryo development in the uterus of a mouse model: *Wnt5a*-dysregulated pregnant mice show increased resorption rates, poor decidual growth, disrupted placental development, embryos were substantially smaller [68].
**rs12138039**	100	8.3e-5	***ARHGEF11***	SNP is a synonymous mutation in a gene that regulates vascular smooth muscle contraction. ARHGEF11 modulates the effects of angiotensin [69], a vasoconstrictive hormone associated with preterm birth [70] likely due to a blood pressure-regulating potency. *ARHGEF11* is also expressed in human myometrium at labour [71]. It obtained the most extreme permutation p-value in a family-based association study of idiopathic preterm birth [72]. Binds to ABCA1.
**rs4789863**	106	8.5e-5	***TIMP2***	SNP is an eQTL for a tissue inhibitor of metalloproteinases TIMP2 [22]. TIMP2 can react to angiogenic factors and directly suppress the proliferation of endothelial cells, thus inhibiting trophoblast invasion and leading to fetal hypoxia [73], intrauterine growth restriction, preeclampsia[74], and consequently preterm birth [75]. Maternal genetic variant in *TIMP2* was associated with spontaneous preterm labor before [76].
**rs2301137**	157	1.2e-4	***GNB3***	SNP is an eQTL for multiple genes, one of which is *GNB3* [33], encoding guanine nucleotide binding protein transducin. A SNP in this gene is associated with essential hypertension; also there is statistical interaction between this SNP, SNP in *ACE* gene (angiotensin I converting enzyme) and hypertension [77].
**rs3746512**	266	2.2e-4	***MMP9***	SNP is an eQTL for extracellular matrix remodeling enzyme matrix metalloproteinase MMP9 [22]. Excess *MMP9* expression (in response to infection/inflammation) may facilitate proteolysis of basement membrane proteins in the extracellular matrix, impede trophoblast invasion in human decidua, impair spiral artery remodeling and reduce uteroplacental blood flow [54].
**rs1457776**	293	2.4e-4	***KCNQ3***	Intronic SNP in gene *KCNQ3* encoding potassium channel. KCNQ3 might be related to angiogenesis during utero-placental vascular development [78]. Expression was significantly upregulated in preeclampsia. a medical condition with structural/functional alterations in placental and maternal vasculature [79].

Genes were selected from the top 284 genes (top 300 SNPs) in maternal GWAS with labor-initiated deliveries. The genes are presented together with the leading SNP from that region and its most extreme empirical *p-value* from three genetic models. *Rank* represents the rank of that SNP among all GWAS results.

#### Cervical insufficiency

Cervical ripening precedes the delivery and allows the fetus to pass through otherwise too-narrow outlet. Two genes described previously might also be involved in cervical ripening, compromising the structural integrity of extracellular matrix too early (**Table 8**).

**Table 8:** An overview of cervical insufficiency genes.

SNP	Index	*p*-value	Gene	Description
**rs4789863**	106	8.5e-5	***TIMP2***	SNP is an eQTL for a tissue inhibitor of metalloproteinases TIMP2 [22]. TIMP2 inhibits protease activity in tissues undergoing remodelling of the extracellular matrix, and can affect cervix dilation, which precedes delivery. Maternal genetic variant in *TIMP2* was associated with spontaneous preterm labor with intact fetal membranes [76], indicating that TIMP2 more likely acts via cervix.
**rs3746512**	266	2.2e-4	***MMP9***	SNP is an eQTL for extracellular matrix remodeling enzyme matrix metalloproteinase MMP9 [22]. MMP9 plays a role in cervical ripening [80]. A genetic variant in *MMP9* promoter is associated with preterm birth [48].

Genes were selected from the top 284 genes (top 300 SNPs) in maternal GWAS with labor-initiated deliveries. The genes are presented together with the leading SNP from that region and its most extreme empirical *p-value* from three genetic models. *Rank* represents the rank of that SNP among all GWAS results.

#### Hormonal imbalance

The fourth group represents three genes that are connected to hormonal problems (**Table 9**), which can lead to preterm birth.

**Table 9:** An overview of hormonal genes.

SNP	Rank	*p*-value	Gene	Description
**rs3117048**	22	2.5e-5	***WNT4***	SNP is located 99 kb from the *WNT4* gene. WNT4 is associated with hyper-androgenism in females (high levels of testosterone, acne, hirsutism) [81], likely due to a mutation increasing androgen biosynthesis [82]. Encodes a signaling protein that is negatively correlated with estrogen and progesterone levels [83], and is associated with uterine hypoplasia [84], as it is a known morphogen controlling uterine changes during pregnancy [83]. Importantly, PTB risk is higher for mothers with polycystic ovary syndrome, notable for high androgen levels [85]. Also, small intrauterine space (uterine hypoplasia) might be causally linked to the shorter gestational age [86].
**rs6718188**	66	5.7e-5	***SP3***	SNP is an LD (0.92 r^2^) with the SNP in 3'-UTR of the gene *SP3*. SP3 mediates progesterone-dependent induction of the hydroxysteroid dehydrogenase gene (involved in production of progesterone and testosterone) in human endometrium [87].
**rs12202611**	138	1.1e-4	***OPRM1***	Proximal gene ***OPRM1*** encodes μ-opioid receptor (MOR). The MOR is the main target of endogenous opioid system [88], which has been implicated in the regulation of hormonal secretion and uterine contractility during pregnancy [89, 90]. Interestingly, *OPRM1* contains an important modern-human-specific variant [91] (gestational in our species is very different from other primates).

Genes were selected from the top 284 genes (top 300 SNPs) in maternal GWAS with labor-initiated deliveries. The genes are presented together with the leading SNP from that region and its most extreme empirical *p-value* from three genetic models. *Rank* represents the rank of that SNP among all GWAS results.

## Discussion

In our study, GWA analyses showed no genome-wide significant associations. However, using a gene-set enrichment analysis of GWA results, we found evidence that genes acting in mothers might contribute to gestational age in deliveries that start with labor. These genes are known for their involvement in processes that affect the duration of gestation (e.g., infection/inflammation).

### Genome-wide association study

Using a standard genome-wide significance threshold of 5×10^−8^ none of the six GWA analyses revealed significant associations. Similarly as in previous study [10], we used two types of study individuals: mothers and children, as the genes affecting pregnancy might manifest via both genomes. We further stratified our analyses based on the type of delivery initiation: deliveries starting with PROM, deliveries that start with labor, and all pregnancies together (**Fig 1**). Instead of dichotomising a continuous phenotype (preterm and term groups), we directly utilised accurately dated (ultrasound-based method) gestational age, retaining phenotypic variability. The long tail of the skewed phenotype distribution was oversampled (**S1 Fig**) to gain more power to detect large effects. The samples used in our study were collected in a single country and represent ethnically homogenous population. We also investigated allelic interactions (dominance effects) that are likely to contribute to the broad-sense heritability estimates of gestational age [7]. Additionally, our study did not set arbitrary minor-allele frequency filters and used permutation-based association tests, which are less affected by phenotypic outliers or small counts in the minor genotypic group. We believe that these analytical aspects supplement the methods of preceding studies [9, 10].

The exploratory nature of our study (2 types of genomes × 3 types of onset of delivery × 3 genetic models) requires adequate corrections for multiple testing. However, as most of the tests are not independent, a simple Bonferroni correction would be overly conservative. We chose to present uncorrected p-values, at the same time cautioning the reader to remember that more statistical tests were done than in a single GWAS.

### Gene-set enrichment analysis

Subsequent gene-set enrichment analyses indicated that one of our GWAS ranked markers in a biologically meaningful manner (**Fig 4**). Two previous GWA studies investigating preterm birth [9, 10] did not provide such evidence. Enrichment in known pregnancy-related genes justifies a closer inspection of top loci (see Literature-informed analyses) and warrants new GWA studies with larger sample sizes.

The results from gene-set enrichment analysis (**Fig 4**) illustrate the advantage of stratifying study subjects based on the onset of delivery. Only the GWAS investigating *mothers* with *labor*-initiated deliveries showed expected enrichment in pregnancy-related gene-sets and no enrichment in control gene-sets. The reasons for this could be that maternal genes play a more important role than the fetal. However, a smaller number of children (1.5-times less than mothers) could also explain this observation. Similarly, GWAS investigating PROM deliveries had a lower statistical power to detect associations (4-times smaller sample size) than GWAS investigating labor-initiated deliveries. Also, genetically determined gestational age in PROM pregnancies is likely to be shortened by environmental factors (e.g., the severity of the microbial invasion of the amniotic cavity), thus introducing noise and reducing the power of GWAS. Interestingly, even though analysis of mixed pregnancies had the largest sample size, it showed low enrichment in pregnancy-related genes. This observation suggests that gestational age determined by two onsets of delivery (labor and PROM) actually represents two separate endophenotypes.

Based on the results from gene-set enrichment analyses, in the literature-informed analyses we chose to investigate only the top SNPs from the *maternal* GWAS in *labor*-initiated deliveries.

### Literature-informed overview of GWAS results

In the seminal publication by Romero et al. [92], the authors summarised the main pathological processes involved in the preterm parturition syndrome: (1) intrauterine infection/inflammation; (2) placental insufficiency (uteroplacental perfusion, angiogenic imbalances, decidualisation); (3) uterine overdistension and contractility; (4) abnormal allograft reaction; (5) allergy; (6) cervical insufficiency; (7) hormonal imbalance. Some genes implicated by the top 300 SNPs from maternal GWAS in labor-initiated deliveries could be comfortably assigned to these processes: infection/inflammation (*NFKB1*, *TLR4*, *IRF8*, *ABCA1*, *TREML2*, *MEFV*, *WNT5A*, *NFKBIA*), placental insufficiency (*ENG*, *TOR2A*, *IGF2*, *KCNQ3*, *GNB3*, *LEFTY2*, *ARHGEF11*, *WNT4*, *WNT5A*), cervical insufficiency (*MMP9*, *TIMP2*), and hormonal imbalance (*WNT4*, *OPRM1*, *SP3*).

We found 32 genes (**Table 4**) that 1) had suggestive evidence of association in GWA analysis, 2) were likely to have their function/expression affected by top GWAS SNPs, 3) had phenotype-relevant biological functions, and 4) their proteins formed clusters of interaction. Most of these genes belong to the "bacterial infection" group (**Table 5**).

Similar future studies might benefit from these observations: inclusion of recessive and dominant genetic models was advantageous, because allelic interactions (dominance effects) implicated approximately 90% of genes with biological relevance (**Table 4**). Similarly, 30% of genes would have been overlooked if a minor-allele frequency filter (MAF > 0.1) were to be applied, and over 50% would have been lost if GWAS sample size were to be increased by adding PROM-delivering mothers (*N* = 336) to the mothers with labor-initiated deliveries (*N* = 1407).

Replication studies should take into account that common infections in various geographical regions and climates might be caused by specific strains/species of bacteria. Similarly, different human populations might be unique in their immunity (vitamin D and sun exposure, vaccination policies, specific hygiene-related behaviours).

Infection/inflammation-related genes from our analyses (**Table 5**) could be used in gene-environment interaction (G×E) studies investigating how genotypes modulate the effect of infection-during-pregnancy on the gestational age at birth. Such studies could create the tools to identify women at high risk for delivering preterm.

## Conclusion

In this study, no genome-wide significant associations with gestational age were found. We highlight 32 genes for the follow-up research, providing suggestive statistical evidence and biological relevance to gestational age, especially via inflammatory-pathways. Our study illustrates how post-GWAS analysis might give insights into the aetiology of the phenotype even without clear GWAS signals.

## Supporting Information

S1 Fig Phenotype distribution is six GWAS analyses. *Frequency* denotes the number of individuals with a particular value of gestational age. The red line represents phenotype distribution in the whole MoBa cohort with same exclusion criteria applied as was for genotyped sample, only without case-oversampling. Maximal height of the red line was adjusted to match the histogram height. Individuals in different histograms might represent the same pregnancy.
(TIFF)

S2 Fig Manhattan plot for fetal GWAS of gestational age in labor-initiated deliveries. In total 884 fetal genomes were used. Each SNP was assigned the most extreme empirical p-value from three genetic models (additive, recessive, dominant). The top line indicates a genome-wide significance level (5×10^−8^), while the bottom line marks a significance level (5×10^−4^) determining the number of “clumps” (independent loci that are used in gene-set enrichment analyses).
(TIF)

S1 File Results from all 6 GWA analyses. *Best_emp_P—*the most extreme empirical p-value from three genetic models, *Eff/Ref—*the effect allele and the reference allele, *Genetic model*—the most significant genetic model for that SNP. Only SNPs with *best_emp_P* values ≤10^−3^ are shown.
(ZIP)

S2 File All genes from 12 pregnancy-related gene-sets. (ZIP)

S1 Table Text-mining PubMed abstracts for pregnancy-related genes. The table shows keywords and their queries used to search PubMed database. Numbers of keyword-related genes are shown before and after filtering.
(XLSX)
