# Correction: Literature-Informed Analysis of a Genome-Wide Association Study of Gestational Age in Norwegian Women and Children Suggests Involvement of Inflammatory Pathways

**DOI:** 10.1371/journal.pone.0165328

**Published:** 2016-10-19

**Authors:** The PLOS ONE Staff

The captions for Tables 1 and 4 were incorrectly incorporated in the manuscript text. The authors have provided the correct tables and captions here. The publisher apologizes for this error.

**Table 1:** Top 20 independent loci from maternal GWAS of gestational age in labor-initiated deliveries.

Chr	BP	SNP	P	E/R	Mod	nEE	nER	nRR	mEE	mER	mRR	Genes
**6**	164389165	rs593254	3.32e-6	A/G	ADD	76	480	851	260	264	268	
**2**	134837980	rs13410504	5.64e-6	G/A	REC	7	237	1163	221	265	267	
**1**	226209989	rs17515010	1.00e-5	G/A	REC	4	141	1261	205	264	266	*SDE2*, *PYCR2*, ***LEFTY2***, *H3F3A*, *H3F3AP4*
**1**	81391541	rs17105699	1.03e-5	G/A	DOM	6	165	1232	273	259	267	
**20**	7618077	rs6086132	1.06e-5	A/G	DOM	162	649	596	269	268	263	
**5**	10501076	rs2589658	1.15e-5	C/A	REC	330	684	393	262	267	267	*ROPN1L*, *ROPN1L-AS1*, *MARCH6*, *ANKRD33B*
**4**	103537442	rs1609798	1.48e-5	A/G	REC	128	601	677	259	266	268	***NFKB1***, *MANBA*
**9**	130417033	rs10117075	1.55e-5	A/G	REC	12	190	1205	237	268	266	*TTC16*, ***TOR2A***, *STXBP1*, *SH2D3C*, *PTRH1*, *FAM129B*
**14**	91352234	rs6575165	1.56e-5	A/G	ADD	87	478	842	260	264	268	*TTC7B*, *RPS6KA5*
**10**	88336279	rs2588278	1.58e-5	A/G	ADD	260	680	467	270	266	264	*WAPAL*, *OPN4*, *LDB3*
**1**	36879232	rs3007217	1.81e-5	G/A	ADD	150	593	664	270	268	264	*STK40*, *SH3D21*, *OSCP1*, *MRPS15*, *LSM10*, *EVA1B*, *CSF3R*
**16**	18067234	rs151699	1.86e-5	C/A	REC	1	133	1272	161	266	266	
**16**	3344618	rs220381	2.12e-5	G/A	DOM	159	559	689	270	268	264	*ZSCAN32*, *TIGD7*, *OR2C1*, *OR1F2P*, *OR1F1*, *MTRNR2L4*, ***MEFV***
**10**	87762136	rs11201867	2.33e-5	A/G	ADD	22	304	1081	275	270	265	*GRID1*
**1**	22345093	rs3117048	2.49e-5	A/G	REC	146	633	628	273	266	265	***WNT4***, *HSPG2*, *CELA3B*, *CELA3A*, *CDC42*
**4**	112524778	rs10015214	2.60e-5	A/G	DOM	312	675	420	266	268	263	
**6**	41164005	rs6915083	2.64e-5	G/A	REC	197	648	561	261	268	266	TREM*, TREML*, *NFYA*, *ADCY10P1*
**16**	85941774	rs305080	2.67e-5	A/G	REC	143	586	678	273	266	265	***IRF8***
**1**	88453303	rs3008465	2.67e-5	C/A	ADD	71	532	802	272	268	264	
**6**	123749752	rs1343962	2.80e-5	A/G	ADD	303	713	390	263	266	269	*TRDN*

Table 1 was pruned to show only independent loci. *BP*—physical position on the chromosome in hg19 coordinates, *P*—the most extreme empirical p-value from three genetic models, *E/R*—the effect allele and the reference allele, *Mod*—the most significant genetic model for that SNP, *nXX*—number of individuals in each genotypic group, *mXX*—mean gestational age in each genotypic group. Interpretation of mean gestational age values should take into account the bimodal phenotype distribution of genotyped individuals (S1 Fig). *Genes* were assigned to SNPs based on a 100 kb offset rule. Asterisk (*) indicates a gene family with multiple genes in that locus. No multiple-test correction is applied. Bolded genes are described in the literature-informed analyses. Genes with unknown function (*LINC*, *LOC* etc.) are not listed.

**Table 4:** Loci of biological relevance from maternal GWAS of gestational age in labor-initiated deliveries.

Rank	SNP	Chr	BP	P	E/R	Mod	nEE	nER	nRR	mEE	mER	mRR	Genes
**5**	rs17515010	1	226209989	1.00e-5	G/A	REC	4	141	1261	205	264	266	*LEFTY2*
**10**	rs1609798	4	103537442	1.48e-5	A/G	REC	128	601	677	259	266	268	*NFKB1*
**11**	rs10117075	9	130417033	1.55e-5	A/G	REC	12	190	1205	237	268	266	*ENG*
**13**	rs2287116	9	130420813	1.55e-5	A/C	REC	12	210	1185	237	267	266	*TOR2A*
**19**	rs220381	16	3344618	2.12e-5	G/A	DOM	159	559	689	270	268	264	*MEFV*
**22**	rs3117048	1	22345093	2.49e-5	A/G	REC	146	633	628	273	266	265	*WNT4*
**24**	rs6915083	6	41164005	2.64e-5	G/A	REC	197	648	561	261	268	266	*TREM1*, *TREML2*, *TREML4*
**25**	rs305080	16	85941774	2.67e-5	A/G	REC	143	586	678	273	266	265	*IRF8*
**41**	rs4312673	3	48401307	3.67e-5	A/G	DOM	1	72	1332	282	256	267	*CAMP*
**65**	rs634335	1	36335862	5.63e-5	C/A	DOM	23	310	1074	266	262	267	*AGO3*
**66**	rs6718188	2	174761611	5.73e-5	A/C	ADD	157	611	638	269	268	264	*SP3*
**75**	rs12336969	9	107679500	6.10e-5	A/C	REC	7	201	1199	229	267	266	*ABCA1*
**88**	rs2177539	7	16652523	7.24e-5	G/A	REC	109	566	728	259	267	267	*ANKMY2*
**98**	rs3913369	3	55481075	8.22e-5	A/C	ADD	69	498	840	262	264	268	*WNT5A*
**100**	rs12138039	1	156918137	8.29e-5	A/G	DOM	6	185	1214	259	261	267	*ARHGEF11*
**101**	rs4075688	3	195848264	8.30e-5	G/A	REC	177	668	559	261	266	268	*TFRC*
**106**	rs4789863	17	76897347	8.52e-5	A/G	DOM	1	122	1281	251	259	267	*TIMP2*
**109**	rs11866271	16	24881152	8.74e-5	C/A	DOM	107	582	713	266	264	268	*TNRC6A*
**117**	rs3021274	22	40395084	9.22e-5	A/G	DOM	230	653	524	269	267	264	*TNRC6B*
**138**	rs12202611	6	154237443	1.08e-4	G/A	REC	7	295	1105	230	266	266	*OPRM1*
**146**	rs395643	14	56541638	1.12e-4	G/A	REC	14	310	1083	242	266	266	*PELI2*
**157**	rs2301137	12	7018949	1.22e-4	A/G	DOM	86	536	784	267	263	268	*GNB3*, *SPSB2*
**173**	rs12435366	14	35838389	1.41e-4	A/G	REC	97	550	745	259	267	266	*NFKBIA*
**197**	rs7045953	9	120485795	1.56e-4	G/A	ADD	37	379	991	272	269	265	*TLR4*
**266**	rs3746512	20	44592636	2.18e-4	A/G	REC	34	394	979	253	266	267	*MMP9*
**284**	rs4849122	2	113560921	2.34e-4	G/A	REC	7	158	1242	233	266	266	*IL1A*, *IL1B*
**293**	rs1457776	8	133360660	2.39e-4	A/G	REC	52	433	922	256	266	267	*KCNQ3*
**299**	rs942364	13	28896097	2.44e-4	A/G	DOM	20	307	1080	270	262	267	*PAN3*

The SNPs were selected from the top 300 GWAS results, based on their proximity and/or functional relationship with genes biologically relevant to gestational age. *Rank*—the rank of that SNP among all GWAS results, based on the most significant empirical p-value (*P*) from three genetic models, *BP*—physical position on the chromosome (*Chr*) in hg19 coordinates, *E/R*—the effect allele and the reference allele, *Mod*—the most significant genetic model for that SNP, *nXX*—number of individuals in each genotypic group, *mXX*—mean of gestational age in each genotypic group. Interpretation of mean gestational age values should take into account the bimodal phenotype distribution of genotyped individuals (**S1 Fig**). No multiple-test correction is applied.
