# Supplementary figures and images for: Literature-Informed Analysis of a Genome-Wide Association Study of Gestational Age in Norwegian Women and Children Suggests Involvement of Inflammatory Pathways
Source: PLoS One. 2016 Aug 4;11(8):e0160335. doi: 10.1371/journal.pone.0160335 (PMC4973994; doi:10.1371/journal.pone.0160335)

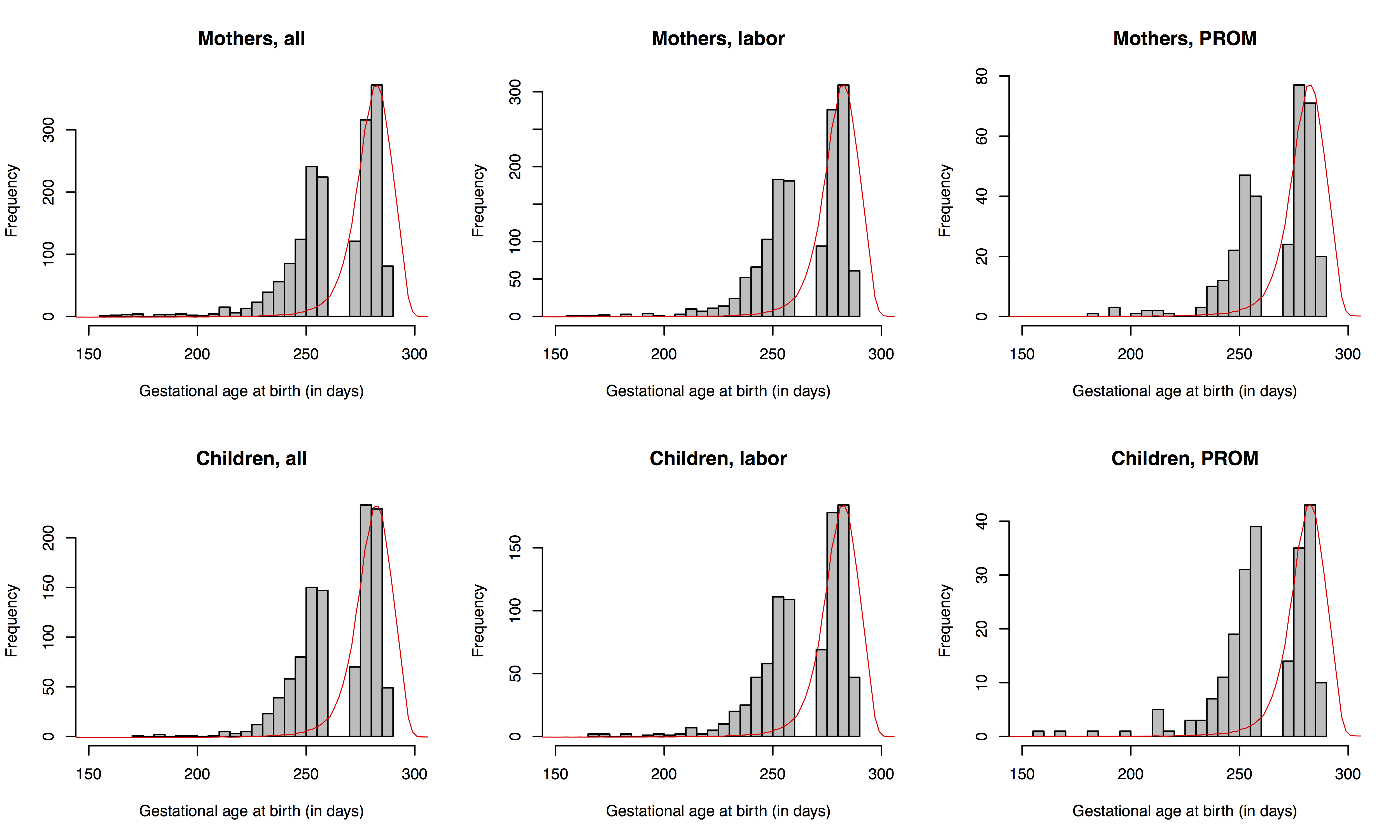

Supplement: S1 Fig — Frequency denotes the number of individuals with a particular value of gestational age. The red line represents phenotype distribution in the whole MoBa cohort with same exclusion criteria applied as was for genotyped sample, only without case-oversampling. Maximal height of the red line was adjusted to match the histogram height. Individuals in different histograms might represent the same pregnancy. (TIFF) [file pone.0160335.s001.tiff]

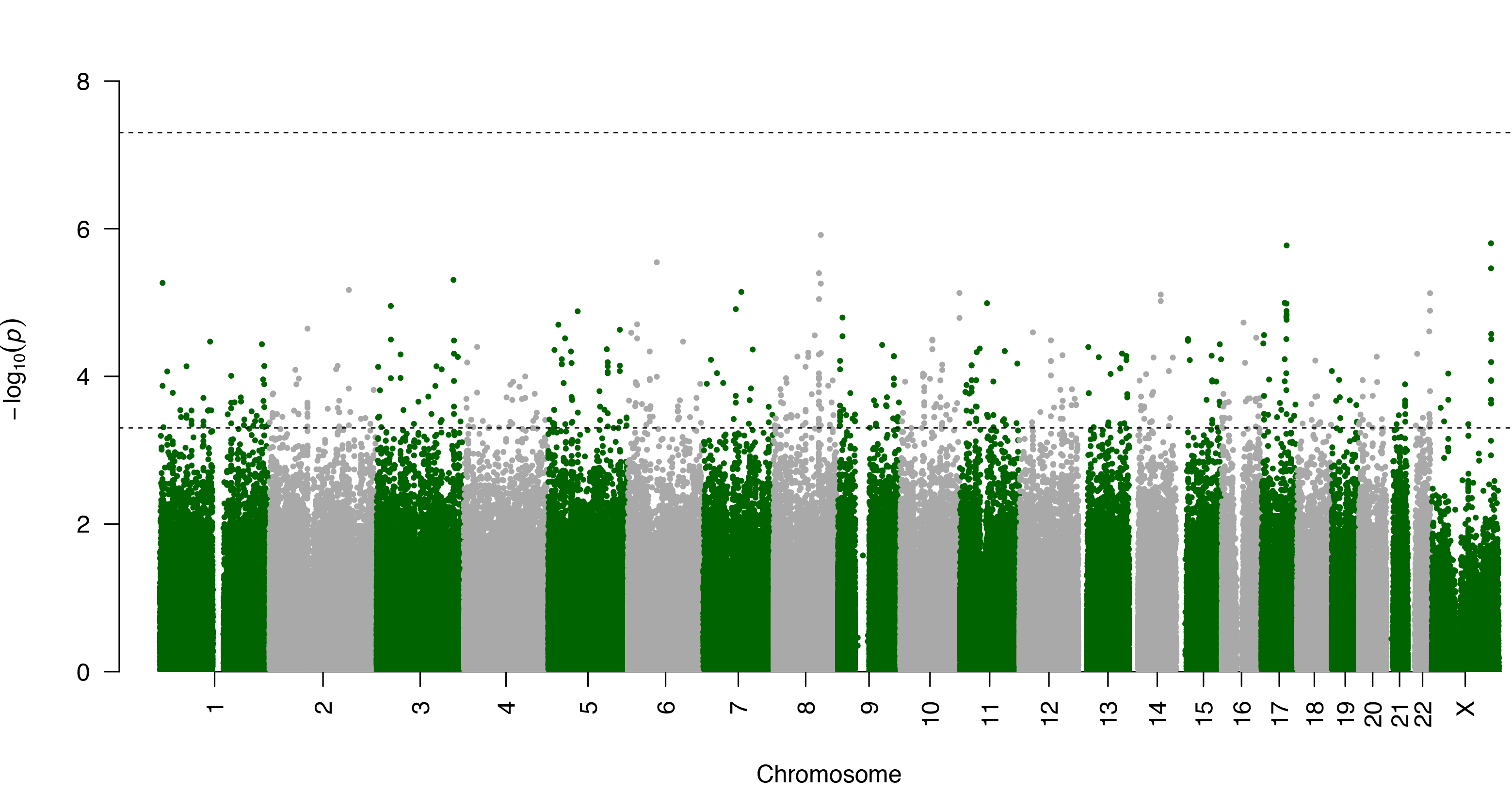

Supplement: S2 Fig — In total 884 fetal genomes were used. Each SNP was assigned the most extreme empirical p-value from three genetic models (additive, recessive, dominant). The top line indicates a genome-wide significance level (5×10^−8^), while the bottom line marks a significance level (5×10^−4^) determining the number of “clumps” (independent loci that are used in gene-set enrichment analyses). (TIF) [file pone.0160335.s002.tif]
